# Factors influencing fatigue, mental workload and burnout among Chinese health care workers during public emergencies: an online cross-sectional study

**DOI:** 10.1186/s12912-024-02070-0

**Published:** 2024-06-25

**Authors:** Qian Xiong, Feng Luo, Yue Chen, Yi Duan, Jie Huang, Hong Liu, Pengjuan Jin, Rong Li

**Affiliations:** The First Affiliated Hospital of Chongqing Medical University, No.1 Youyi Road, Yuzhong District, Chongqing, 400042 China

**Keywords:** Public emergencies, Health care workers, Fatigue, Mental workload, Burnout

## Abstract

**Objectives:**

The purpose of this study was to investigate fatigue, mental workload, and burnout among health care workers (HCWs) and explore the possible underlying factors.

**Materials and methods:**

An online cross-sectional survey design was used to collect data from HCWs in Chongqing, China. The online survey included the Fatigue Severity Scale, NASA Task Load Index, and Chinese version of the Maslach Burnout Inventory-General Survey to assess fatigue, mental workload, and burnout, respectively, and was conducted from February 1 to March 1, 2023.

**Results:**

In this study, the incidence of fatigue and burnout among HCWs was 76.40% and 89.14%, respectively, and the incidence of moderate to intolerable mental workloads was 90.26%. Work–family conflict, current symptoms, number of days of COVID-19 positivity, mental workload, burnout and reduced personal accomplishment were significantly associated with fatigue. Mental workload was affected by fatigue and reduced personal accomplishment. Furthermore, burnout was influenced by marital status and fatigue. Moreover, there was a correlation among mental workload, fatigue, and burnout.

**Conclusions:**

Fatigue, mental workload and burnout had a high incidence and were influenced by multiple factors during COVID-19 public emergencies in China.

## Introduction

Public health emergencies often trigger psychological distress among individuals and communities due to their sudden, urgent, and serious nature, as well as the high level of uncertainty they cause [1]. Due to their unique work environment, high levels of stress, and increased risk of infection, frontline healthcare workers (HCWs) who provide care and services to sick people often experience mental health issues during public health emergencies [2, 3]. They may experience symptoms such as fatigue, high mental workload, and burnout, which have negative consequences such as medical errors, poor quality of care, and increased patient mortality [4–6].

Fatigue primarily manifests as physical and mental exhaustion, including reduced concentration and motivation [7, 8]. Fatigue has become common among HCWs during the COVID-19 pandemic, with a moderate to high prevalence of 35.06–72.2% [9, 10]. Cardiopulmonary symptoms, psychiatric symptoms, and muscle weakness may also occur following COVID-19 infection [11], facilitating the physical exhaustion of HCWs. In addition, limited social interactions due to isolation measures and different viral transmission routes have led to mental fatigue among HCWs. All of these factors exacerbate their symptoms of fatigue, resulting in declines in work quality and poor patient outcomes [12, 13].

Moreover, previous studies have shown that the occurrence of an epidemic adversely affects the mental health of HCWs, including the loss of self-confidence and inability to make decisions [14, 15]. The occurrence of these factors affects the mental workload of HCWs. Mental workload is defined as the weight, cost, and quantity of effort needed to complete occupational tasks, referring to the ability to process information, make clinical decisions, and communicate with patients and their families [16–18]. A high mental workload contributes to fatigue, decreased efficiency, poor performance, and increased patient mortality [19–21].

Furthermore, more than 50% of HCWs experience severe fatigue and have a heavy mental workload, which can lead to burnout [22]. Maslach and Jackson defined burnout as a reaction to chronic and long-term stress in the workplace characterized by three aspects: emotional exhaustion (EE), depersonalization (DP), and reduced personal accomplishment (RPA) [23]. Notably, burnout generally increases over time [24]. From the peak of the Wuhan COVID-19 epidemic to the strict zero-COVID-19 policy period, the overall incidence of burnout decreased slightly from 51.7 to 50.4% [22, 25], indicating that the overall incidence of burnout among HCWs remained high. Chronic burnout can lead to physical or psychological issues, poor quality of care, medical malpractice, and increased organizational costs [26–28]. However, the incidence of burnout after the strict zero-COVID-19 policies were relaxed is not known, but the reality is not promising.

We hypothesized that the incidence of fatigue, mental workload, and burnout among HCWs was high and that HCWs were strongly affected by the COVID-19 pandemic. Therefore, this study aimed to investigate fatigue, mental workload, and burnout among HCWs and explore their interrelationships. Understanding the mental health of HCWs in this ongoing situation will help us develop better coping mechanisms.

## Materials and methods

### Study design and participants

An online cross-sectional study of HCWs was conducted from February 1 to March 1, 2023. The inclusion criteria were as follows: (1) individuals who provided consent to participate in this study and (2) HCWs, including registered doctors, nurses, and technicians. HCWs who were unwilling to participate in this study were excluded.

### Materials

#### Demographic data questionnaires

Individual and occupational characteristic data were self-reported by the participants and included age, sex, marital status, education level, profession, hospital level, hospital department, work year, intensity of work, work pressure, and work-family conflict. The following data related to personal virus infection were collected: COVID-19 infection status, current symptoms, number of days of COVID-19 positivity, and number of days until recovery from COVID-19.

#### Fatigue severity scale (FSS)

The FSS, which was developed in 1989 by Krupp [29, 30], is mainly used to assess the severity of fatigue. The scale consists of 9 items scored using a 7-point Likert scale ranging from 1 (strongly disagree) to 7 (strongly agree). The total score ranges from 9 to 63 points, with higher scores indicating greater fatigue. A score of 36 points is the threshold, with a score < 36 points indicating no fatigue and a score ≥ 36 points indicating the presence of fatigue [31]. The Cronbach’s α for the FSS in the present study was 0.940.

#### NASA Task load index (NASA-TLX)

Mental workload was assessed with the NASA-TLX. The NASA-TLX, which was designed in the 1970s, was originally developed to measure workload stress among aerospace workers and consists of six dimensions: mental demand, physical demand, temporal demand, performance, effort and frustration [32, 33]. Each dimension is represented by a straight line on a 20-point scale ranging from 0 to 100, and a higher score indicates a greater mental workload [34]. Mental workload was classified based on the NASA-TLX score as follows: scores of 0–20, low; scores of 21–40, mild; scores of 41–60, moderate; scores of 61–80, high; and scores of 81–100, intolerable [35]. The Cronbach’s α in this study was 0.826.

#### The Chinese version of the Maslach Burnout Inventory-General Survey (MBI-GS)

The Chinese version of the MBI-GS was used to measure burnout and has three dimensions: the EE, DP and RPA [36]. The RPA items are reverse scored. Each item is rated on a 7-point Likert scale ranging from 0 (never) to 6 (every day). According to the evaluation criteria of Li et al., burnout was classified as no burnout (all 3 dimension scores below the threshold), mild burnout (any 1 dimension score above the threshold), moderate burnout (any 2 dimension scores above the threshold), or high burnout (all 3 dimension scores above the threshold), using an EE score > 25 points, a DP score > 11 points, and an RPA score > 16 points as the thresholds [37]. The MBI-GS had a Cronbach’s α of 0.863 for burnout in this study.

### Data collection

Owing to the rapid spread of the virus since the relaxation of restrictions, an online questionnaire was created and distributed to HCWs via WeChat, one of the most widely used social media platforms in China. The survey was conducted from February 1 to March 1, 2023. Each phone IP address could be used only once to open and complete the survey to avoid duplication.

### Data analysis

The data were analyzed using IBM SPSS software version 24.0. Quantitative data are presented as the mean ± standard deviation (SD), and qualitative data are presented as frequencies and percentages. Demographic and personal viral infection characteristics associated with fatigue, mental workload, and burnout were examined using t tests and one-way analysis of variance (ANOVA). Pearson’s correlation analysis was used to investigate the relationships among burnout, fatigue, and mental workload. The independent variables with statistical significance (*P* < 0.05) in the univariate analysis and Pearson correlation analysis were included in the multiple linear regression analysis, with fatigue, mental workload, and burnout as the dependent variables. In multiple regression analysis, independence was tested with the Derbin-Watson (D-W) residual test, homogeneity of variance was tested by a scatter plot, and normality was checked with a histogram combined with a normal P-P plot. A p value < 0.05 was considered to indicate a significant difference.

### Ethical consideration

Ethics approval for this study was obtained from the Ethics Committee of the First Affiliated Hospital of Chongqing Medical University (Approval number: K2023-061). Participants read and signed an informed consent form before they started filling out the questionnaire.

## Results

### Descriptive characteristics

A total of 267 completed questionnaires were collected in this study. The majority of participants were female (92.13%), were married (59.18%), were nurses (87.26%), had an undergraduate degree or above (86.14%), came from Grade A hospitals (90.64%), worked in surgical units (47.19%), and had worked for less than 5 years (40.82%). Most participants experienced moderate or higher levels of work intensity (94.38%), work pressure (93.63%), and work-family conflict (75.66%). Regarding personal COVID-19 infection characteristics, most participants had a COVID-19 positivity period of 1–10 days (83.00%) and a recovery period of 11 days or more (84.69%). Moreover, most participants had one or more symptoms (69.66%). More detailed information about the individual, occupational, and COVID-19 infection characteristics of the participants is shown in Tables 1 and 2.

**Table 1:** Univariate analysis of fatigue, mental workload, and burnout in relation to categorical variables(*n* = 267)

Variables		*N*(%)	Fatigue	Mental workload	Burnout			
					Overall	EE	DP	RPA
Age	< 30	120(44.94%)	43.01 ± 12.51	66.03 ± 15.87	51.86 ± 13.28	20.35 ± 6.99	19.06 ± 6.46	12.45 ± 5.61
	30–40	115(43.07%)	45.48 ± 13.48	66.33 ± 16.96	46.57 ± 12.26	18.70 ± 6.68	16.60 ± 5.72	11.27 ± 5.98
	41-	32(11.99%)	40.66 ± 14.76	60.72 ± 23.78	40.81 ± 12.37	15.97 ± 6.62	15.00 ± 5.98	9.84 ± 7.71
F/F’			2.049	0.789	11.260	5.589	7.863	2.263
P			0.131	0.458	0.000	0.004	0.000	0.111
Education	Tertiary and below	37(13.86%)	40.49 ± 12.22	60.15 ± 18.65	45.08 ± 13.79	16.59 ± 6.33	16.70 ± 5.06	11.78 ± 6.51
	Undergraduate degree	172(64.42%)	44.45 ± 13.71	66.62 ± 18.08	47.38 ± 12.80	18.98 ± 6.92	17.13 ± 6.27	11.27 ± 6.39
	Master and above	58(21.72%)	43.93 ± 12.43	65.67 ± 14.17	52.90 ± 13.18	21.14 ± 6.84	19.16 ± 6.67	12.60 ± 4.72
F/F’			1.370	2.110	5.175	5.102	2.661	1.425
P			0.256	0.123	0.006	0.007	0.072	0.246
Profession	Nurse	233(87.26%)	43.92 ± 13.09	73.82 ± 8.55	47.52 ± 12.38	18.89 ± 6.68	17.18 ± 6.02	11.44 ± 6.13
	Doctor	17(6.37%)	48.53 ± 10.78	65.40 ± 17.65	62.88 ± 15.18	25.76 ± 6.57	24.06 ± 6.99	13.06 ± 4.37
	Technician/Other	17(6.37%)	37.29 ± 16.00	58.81 ± 18.95	43.82 ± 14.09	15.59 ± 6.92	15.47 ± 4.89	12.76 ± 7.01
F/F’			3.186	7.771	12.757	10.902	11.369	0.873
P			0.043	0.002	0.000	0.000	0.000	0.419
Hospital level	Grade A	242(90.64%)	44.05 ± 13.25	66.03 ± 17.55	48.24 ± 13.34	19.23 ± 6.99	17.53 ± 6.19	11.48 ± 6.10
	Grade B	15(5.62%)	41.20 ± 14.18	61.07 ± 13.91	50.87 ± 13.62	19.07 ± 6.94	18.33 ± 7.71	13.47 ± 4.88
	Grade C	10(3.74%)	41.40 ± 12.94	59.78 ± 19.61	44.80 ± 9.43	16.40 ± 5.36	15.80 ± 5.65	12.60 ± 7.50
F			0.493	1.133	0.632	0.800	0.504	0.887
P			0.611	0.324	0.532	0.450	0.605	0.413
hospital department	Internal Medicine	57(21.35%)	43.98 ± 12.00	67.11 ± 12.62	49.05 ± 13.80	20.19 ± 6.84	18.23 ± 6.20	10.63 ± 5.45
	Surgery	126 (47.19%)	43.37 ± 13.48	64.30 ± 19.94	46.95 ± 13.10	18.34 ± 6.61	17.17 ± 5.99	11.44 ± 6.06
	Emergency and ICU	23 (8.61%)	50.30 ± 11.50	74.80 ± 13.68	57.13 ± 12.19	23.91 ± 7.39	21.39 ± 6.97	11.83 ± 6.31
	Other	61(22.85%)	42.02 ± 14.10	63.06 ± 16.18	46.87 ± 12.21	17.90 ± 6.75	16.10 ± 6.02	12.87 ± 6.55
F/F’			2.291	4.215	4.309	5.550	4.544	1.406
P			0.079	0.008	0.005	0.001	0.004	0.241
Work year	Less than 5 years	109(40.82%)	43.10 ± 12.40	67.00 ± 14.88	52.29 ± 12.76	20.36 ± 6.84	18.94 ± 6.29	13.00 ± 5.41
	5–10 years	60 (22.47%)	44.20 ± 14.74	63.92 ± 18.90	47.00 ± 13.72	18.65 ± 7.43	17.32 ± 6.19	11.03 ± 6.66
	11–15 years	59(22.10%)	44.75 ± 12.87	65.61 ± 16.97	45.32 ± 12.46	18.49 ± 6.47	16.36 ± 6.16	10.47 ± 5.96
	More than 15 years	39 (14.61%)	43.64 ± 14.20	63.70 ± 22.30	43.36 ± 12.05	17.31 ± 6.70	15.59 ± 5.67	10.46 ± 6.63
F/F’			0.219	0.544	6.727	2.335	3.931	3.302
P			0.883	0.653	0.000	0.074	0.009	0.021
Intensity of work	Low	15(5.62%)	39.27 ± 16.87	52.28 ± 24.82	49.53 ± 14.28	19.87 ± 9.07	18.87 ± 8.07	10.80 ± 7.48
	Medium	140 (52.43%)	39.81 ± 13.40	60.49 ± 17.68	43.49 ± 11.72	16.16 ± 5.53	15.37 ± 5.16	11.96 ± 6.09
	High	112 (41.95%)	49.37 ± 10.33	73.58 ± 11.85	54.05 ± 12.59	22.71 ± 6.51	20.01 ± 6.31	11.33 ± 5.93
F/F’			20.961	27.364	23.288	35.562	19.751	0.475
P			0.000	0.000	0.000	0.000	0.000	0.622
Work pressure	Low	17(6.37%)	36.41 ± 18.46	46.09 ± 25.09	46.53 ± 14.04	17.12 ± 9.30	17.53 ± 8.39	11.88 ± 8.28
	Medium	127(47.56%)	40.17 ± 12.48	61.02 ± 17.10	43.36 ± 11.77	16.13 ± 5.21	15.32 ± 4.85	11.91 ± 5.97
	High	123 (46.07%)	48.54 ± 11.58	72.86 ± 12.35	53.55 ± 12.60	22.47 ± 6.65	19.78 ± 6.44	11.30 ± 5.90
F/F’			16.494	26.133	21.611	34.661	18.831	0.330
P			0.000	0.000	0.000	0.000	0.000	0.719
Work-family conflict	Low	65(24.34%)	37.72 ± 14.30	58.15 ± 20.85	45.80 ± 11.91	16.74 ± 6.65	16.23 ± 6.02	12.83 ± 6.32
	Medium	137 (51.31%)	43.24 ± 12.25	65.04 ± 15.78	46.58 ± 12.39	18.15 ± 6.02	16.59 ± 5.45	11.84 ± 5.84
	High	65(24.34%)	51.02 ± 10.80	73.91 ± 13.29	54.26 ± 14.49	23.54 ± 7.13	20.74 ± 7.00	9.98 ± 6.12
F/F’			18.774	15.608	9.501	21.185	9.946	3.796
P			0.000	0.000	0.000	0.000	0.000	0.024
COVID-19 infection status	Negtive	49(18.35%)	44.12 ± 15.40	69.39 ± 17.25	48.27 ± 14.39	19.51 ± 7.59	17.59 ± 6.42	11.16 ± 6.69
	Positive	4(1.50%)	40.25 ± 21.00	50.21 ± 31.79	39.50 ± 12.77	12.25 ± 2.63	15.25 ± 3.20	12.00 ± 8.49
	Recover from COVID−19	210 (78.65%)	43.71 ± 12.68	65.00 ± 17.08	48.57 ± 12.89	19.17 ± 6.74	17.58 ± 6.30	11.82 ± 5.85
	Rebound positivity	4(1.50%)	47.50 ± 10.88	60.96 ± 18.40	40.50 ± 16.11	18.25 ± 9.91	15.50 ± 4.65	6.75 ± 9.00
F/F’			0.210	2.002	1.084	1.392	0.320	1.029
P			0.889	0.114	0.356	0.245	0.811	0.380
Current symptoms	None	81(30.34%)	39.54 ± 14.59	61.31 ± 20.59	47.02 ± 12.33	17.98 ± 7.12	16.70 ± 6.23	12.35 ± 6.41
	One	61(22.85%)	43.66 ± 12.86	66.95 ± 16.90	46.08 ± 13.43	18.20 ± 6.69	17.20 ± 5.80	10.69 ± 5.98
	Two	45(16.85%)	44.49 ± 13.53	62.01 ± 14.59	48.58 ± 13.42	19.91 ± 6.68	17.56 ± 6.00	11.11 ± 6.31
	More than three	80(29.96%)	47.80 ± 10.69	70.66 ± 14.44	50.99 ± 13.59	20.53 ± 6.88	18.55 ± 6.70	11.91 ± 5.70
F/F’			5.516	5.280	1.951	2.426	1.242	1.026
P			0.001	0.002	0.122	0.066	0.295	0.382

*Abbreviations* EE-Emotional Exhaustion; DP-Depersonalization; RPA- Reduced Personal Accomplishment

**Table 2:** Univariate analysis of fatigue, mental workload, and burnout in relation to categorical variables(*n* = 267)

Variables		*N*(%)	Fatigue	Mental workload	Burnout			
					Overall	EE	DP	RPA
Gender	Male	21(7.87%)	41.19 ± 14.40	66.89 ± 16.33	52.29 ± 13.32	21.05 ± 7.48	19.05 ± 4.78	12.19 ± 5.54
	Female	246(92.13%)	44.01 ± 13.17	65.40 ± 17.58	47.91 ± 13.18	18.95 ± 6.87	17.38 ± 6.35	11.58 ± 6.14
t			−0.936	0.374	1.457	1.332	1.173	0.439
P			0.350	0.709	0.146	0.184	0.242	0.661
marital status	Married	158(59.18%)	43.91 ± 13.60	65.06 ± 18.49	45.03 ± 12.03	17.86 ± 6.51	16.34 ± 5.62	10.83 ± 6.34
	Unmarried/other	109(40.82%)	43.61 ± 12.83	66.18 ± 15.93	52.94 ± 13.53	20.94 ± 7.14	19.21 ± 6.74	12.79 ± 5.53
t/t’			0.179	−0.527	−5.014	−3.644	−3.654	−2.679
P			0.858	0.599	0.000	0.000	0.000	0.008
the number of days of COVID-19 positivity ^a^	1–10	166(83.00%)	42.99 ± 12.88	65.65 ± 16.96	47.58 ± 13.50	18.76 ± 7.11	17.44 ± 6.46	11.39 ± 5.97
	11-	34(17.00%)	47.91 ± 12.33	63.23 ± 17.98	48.94 ± 11.19	19.18 ± 5.46	17.29 ± 5.32	12.47 ± 5.68
t/t’			-2.043	0.750	-0.549	−0.323	0.123	−0.973
P			0.042	0.454	0.584	0.747	0.902	0.332
number of days until recovery from COVID-19 ^b^	1–10	30(15.31%)	48.17 ± 10.43	71.53 ± 11.00	50.40 ± 16.29	20.97 ± 7.78	19.53 ± 7.45	9.90 ± 6.72
	11-	166(84.69%)	43.06 ± 13.15	64.13 ± 17.77	47.36 ± 12.48	18.45 ± 6.63	17.04 ± 5.98	11.86 ± 5.74
t/t’			2.014	3.050	1.172	1.865	2.024	−1.683
P			0.045	0.003	0.243	0.064	0.044	0.094

**a**:COVID-19 positive days exclud COVID-19 Negtive patients 49, the missing 18. **b**: days until recovery from COVID-19 exclud COVID-19 Negtive patients 49, COVID-19 Positive patients 4, the missing 18. Abbreviations: EE, emotional exhaustion; DP, depersonalization; RPA, reduced personal accomplishment

### Univariate analysis results

#### Fatigue

Our results indicated that the prevalence of fatigue was 76.40%, and the mean (SD) fatigue score was 43.79 ± 13.26, as shown in Table 3. Tables 1 and 2 show that profession, intensity of work, work pressure, work-family conflict, current symptoms, number of days of COVID-19 positivity, and number of days until recovery from COVID-19 were correlated with fatigue (*P* < 0.05). As hypothesized, the incidence of fatigue was high and affected by COVID-19 infection.

**Table 3:** Descriptive statistics for fatigue, mental workload, and burnout(*n* = 267)

Variables		*N*(%)	Mean ± SD
Fatige	Overall		43.79 ± 13.26
	≥ 36	204(76.40%)	49.51 ± 8.50
	<36	63(23.60%)	25.27 ± 7.87
Mental workload	Overall		65.52 ± 17.46
	Low	7(2.62%)	13.10 ± 4.46
	Slight	19(7.12%)	35.38 ± 3.46
	Moderate	63(23.60%)	52.58 ± 5.75
	High	133(49.81%)	71.19 ± 5.52
	Intolerable	45(16.85%)	87.76 ± 5.06
	Mental demand		66.10 ± 22.74
	Physical demand		67.87 ± 23.63
	Temporal demand		71.52 ± 21.82
	Performance		60.40 ± 27.48
	Effort		71.72 ± 20.17
	Frustration		55.52 ± 26.70
Burnout	Overall		48.26 ± 13.22
	Zero burnout	29(10.86%)	30.38 ± 7.58
	Slight burnout	154(57.68%)	44.98 ± 8.88
	Moderate burnout	76(28.46%)	59.13 ± 9.97
	High burnout	8(3.00%)	72.88 ± 9.49
Emotional exhaustion	Overall		19.12 ± 6.93
	>25	48(17.98%)	30.52 ± 3.20
	≤ 25	219(82.02%)	16.62 ± 4.64
Depersonalization	Overall		17.51 ± 6.25
	>11	227(85.00%)	18.87 ± 5.76
	≤ 11	40(15.00%)	9.83 ± 1.72
Reduced personal accomplishment	Overall		11.63 ± 6.09
	>16	54(20.22%)	19.48 ± 2.95
	≤ 16	213(79.78%)	9.64 ± 4.97

#### Mental workload

Seven participants (2.62%) had a low mental workload, 19 participants (7.12%) had a light mental workload, 63 participants (23.60%) had a moderate mental workload, 133 participants (49.81%) had a high mental workload, and 45 participants (16.85%) had an intolerable mental workload. The mean (SD) mental workload score was 65.52 ± 17.46, as shown in Table 3. The highest to lowest scores were obtained for the dimensions of effort, temporal demand, physical demand, mental demand, perceived performance and frustration, respectively. Profession, hospital department, intensity of work, work pressure, work-family conflict, current symptoms, and number of days until recovery from COVID-19 were significantly associated with mental workload (*P* < 0.05), as shown in Tables 1 and 2. As assumed, mental workload was significantly prevalent and impacted by contracting COVID-19.

#### Burnout

Of the 267 participants, 29 (10.86%) had no burnout, 238 (89.14%) had burnout, 154 (57.68%) had mild burnout, 76 (28.46%) had moderate burnout, and 8 (3.00%) had severe burnout. As hypothesized, the incidence of burnout was high. Moreover, the percentages of participants with EE, DP, and RPA scores above the cut-offs were 17.98%, 85.00%, and 20.22%, respectively, as shown in Table 3.

We analyzed the features of burnout, which are summarized in Tables 1 and 2. We found that age, marital status, education level, profession, hospital department, work year, intensity of work, work pressure, and work-family conflict were significantly associated with burnout (*P* < 0.05). Contrary to the hypothesis, burnout was not affected by COVID-19 infection. Furthermore, age, marital status, education level, profession, hospital department, intensity of work, work pressure, and work-family conflict were significantly correlated with EE (*P* < 0.05). Age, marital status, profession, hospital department, work year, intensity of work, work pressure, work-family conflict and the number of days until recovery from COVID-19 were associated with the DP score (*P* < 0.05). Additionally, marital status, work year, and work-family conflict were related to RPA (*P* < 0.05).

### Relationships among fatigue, mental workload and burnout

As shown in Table 4, the correlation analysis revealed that fatigue and mental workload were associated with burnout (overall: *r* = 0.514, *P* < 0.01; overall: *r* = 0.264, *P* < 0.01).

**Table 4:** Correlation analysis of burnout in relation to fatigue and mental workload(*n* = 267)

Variables	1	2	3	4	5	6
Burnout	1					
EE	0.877**	1				
DP	0.805**	0.794**	1			
RPA	0.347**	−0.050	−0.184**	1		
Fatigue	0.514**	0.626**	0.534**	−0.145*	1	
Mental workload	0.264**	0.363**	0.307**	−0.155*	0.395**	1

**P< 0.01, *P< 0.05

**Table 5:** Multiple linear regression analysis of fatigue, mental workload, and burnout (*n* = 196)

Model	Independent variables	B	SE	t	*P*	95%CI	
						Lower	Upper
Fatigue							
	(Constant)	13.221	7.535	1.755	0.081	−1.644	28.086
	Profession	−1.793	1.504	−1.192	0.235	−4.761	1.174
	Intensity of work	0.500	1.921	0.261	0.795	−3.290	4.291
	Work pressure	0.515	1.981	0.260	0.795	−3.393	4.423
	Work-family conflict	2.456	1.201	2.044	0.042	0.086	4.826
	Current symptoms	1.256	0.631	1.991	0.048	0.011	2.500
	The number of days of COVID-19 positivity	4.578	1.898	2.412	0.017	0.833	8.322
	Number of days until recovery from COVID-19	−1.538	1.969	−0.781	0.436	−5.422	2.346
	Mental workload	0.116	0.050	2.340	0.020	0.018	0.214
	Burnout	0.558	0.193	2.886	0.004	0.177	0.939
	DP	−0.273	0.363	−0.751	0.453	−0.989	0.443
	RPA	−0.621	0.255	−2.435	0.016	−1.124	−0.118
Mental workload							
	(Constant)	44.995	10.165	4.427	0.000	24.941	65.050
	Profession	−2.240	2.424	−0.924	0.357	−7.023	2.543
	hospital department	0.278	0.773	0.359	0.720	−1.247	1.802
	Intensity of work	1.248	2.834	0.441	0.660	−4.342	6.839
	Work pressure	5.248	2.896	1.812	0.072	−0.466	10.962
	Work-family conflict	0.427	1.790	0.239	0.812	−3.104	3.958
	Current symptoms	1.781	0.934	1.907	0.058	−0.061	3.623
	Number of days until recovery from COVID-19	−3.962	2.879	−1.376	0.170	−9.641	1.717
	Fatigue	0.220	0.106	2.070	0.040	0.010	0.429
	EE	0.532	0.288	1.847	0.066	−0.036	1.101
	DP	−0.159	0.284	−0.560	0.576	−0.719	0.401
	RPA	−0.393	0.185	−2.118	0.036	−0.758	−0.027
Burnout							
	(Constant)	9.346	8.194	1.141	0.256	−6.821	25.513
	Age	−0.691	2.334	−0.296	0.768	−5.295	3.913
	Marital status	6.912	2.187	3.160	0.002	2.597	11.226
	Education	2.112	1.284	1.645	0.102	−0.421	4.645
	Profession	−0.495	1.896	−0.261	0.794	−4.235	3.246
	hospital department	0.034	0.576	0.058	0.954	−1.102	1.169
	Work year	−0.476	1.431	−0.333	0.740	−3.300	2.348
	Intensity of work	1.678	2.136	0.786	0.433	−2.536	5.892
	Work pressure	0.942	2.165	0.435	0.664	−3.329	5.213
	Work-family conflict	0.947	1.333	0.710	0.479	−1.683	3.577
	Fatigue	0.436	0.071	6.128	0.000	0.296	0.576
	Mental workload	0.013	0.054	0.233	0.816	−0.093	0.118

Model performance of Fatigue: R^2^ = 0.471,adjusted R^2^ = 0.440,F = 14.913,*P* = 0.000

Model performance of Mental workload: R^2^ = 0.333,adjusted R^2^ = 0.293,F = 8.358,*P* = 0.000

Model performance of Burnout: R^2^ = 0.389,adjusted R^2^ = 0.352,F = 10.650,*P* = 0.000

### Multiple linear regression analysis results

The model including fatigue had an R^2^ of 0.471 and an adjusted R^2^ of 0.440. Variables that were significantly associated with greater fatigue included experiencing more frequent work-family conflict (t = 2.044, *P* = 0.042), having a greater number of current symptoms (t = 1.991, *P* = 0.048), having a greater number of days of COVID-19 positivity (t = 2.412, *P* = 0.017), having a greater mental workload (t = 2.340, *P* = 0.020), and experiencing increased burnout (t = 2.886, *P* = 0.004). Decreased fatigue was significantly associated with RPA (t=-2.435, *P* = 0.016).

The mental workload model had an R^2^ of 0.333 and an adjusted R^2^ of 0.293. These variables were significantly associated with more fatigue (t = 2.070, *P* = 0.040). Decreased mental workload was significantly associated with RPA (t=-2.118, *P* = 0.036).

The model of burnout had an R^2^ of 0.389 and an adjusted R^2^ of 0.352. Variables that were significantly associated with marital status were unmarried status (t = 3.160, *P* = 0.002) and increased fatigue (t = 6.128, *P* = 0.000).

## Discussion

This study surveyed 267 HCWs after the strict zero-COVID-19 policies were relaxed and found that they experienced high levels of fatigue, mental workload, and burnout. Our analysis showed that many factors affected the fatigue, mental workload, and burnout of HCWs, and that these variables are correlated.

Our 76.40% fatigue incidence rate was higher than that in a previous study, which reported an incidence ranging from 26.6 to 41.2% at the start of the COVID-19 pandemic [38, 39]. Our mean (SD) fatigue score was 43.79 ± 13.26. The reason for the high prevalence of fatigue is that the HCWs were both patients and health caregivers. Our study corroborated the previously discussed associations among work-family conflict, mental workload, burnout and fatigue,as shown in Table 5. Notably, we found that greater numbers of current symptoms and days of COVID-19 positivity were significantly associated with fatigue [40–42]. Patients with acute COVID-19 can develop neurological and psychiatric symptoms during and after the acute phase of illness [43–45]. These chronic symptoms made the HCWs more prone to fatigue. Therefore, it is necessary to pay attention to changes in HCWs’ physical condition caused by COVID-19 through methods such as electrocardiography and CT. In addition, in a previous study, one-third of HCWs experienced residual symptoms even after returning to work, with persistent fatigue being a common symptom [46, 47]. This prompted HCWs to rationalize their work in accordance with their physical status, especially the presence of current symptoms and number of days of COVID-19 positivity. Moreover, fatigue is not only a state but also a process that can gradually lead to worsening fatigue symptoms [9]. This could increase the risk of poor clinical decision-making and compromise patient safety [26]. Therefore, early identification of fatigue and timely intervention are necessary Table 5.

We also found that RPA was related to a reduction in fatigue and mental workload. RPA is characterized by feelings of incompetence and a lack of achievement and productivity at work [23]. Kakemam reported that RPA was associated with the risk of medical errors and verbal abuse by patients and their families [48]. Alleviation of RPA reduced the occurrence of adverse events and reduced work and psychological stress among HCWs, which may be one of the reasons why RPA was related to fatigue and mental workload in our study. Therefore, skills training, further education, and professional development to improve professional achievement at work are essential.

In our study, the incidence of a moderate to intolerable mental workload was 90.26%, higher than the pooled incidence of 54% reported in the study of Yuan et al. [49]. This was due to the suspension of medical activities to treat patients infected with COVID-19, exposing medical and surgical staff to a new complex work environment, causing them to face new challenges. A total of 23.9% of the respondents in the study by González-Gil et al. noted greater clinical autonomy in decision-making during the COVID-19 pandemic than before the pandemic [50]. This placed greater demands on HCWs and increased the burden of their mental workload. Costin et al. reported flexible working, time management, and team support increased efficiency, productivity, and creativity [51]. Telehealth utilization was used to provide healthcare services during the pandemic [52]. Flexible working arrangements and clinical support, including remote technologies, are effective ways to alleviate HCWs’ mental workload.

In our study, the mean mental workload score was 65.52 ± 17.46. Additionally, effort and frustration had the highest and lowest scores, respectively, which was consistent with the study of Sarsangi et al. [53]. Nikeghbal et al. reported that nurses who were assessed for mental workload by the NASA-TLX had the highest perceived performance scores and the lowest frustration scores [54]. In Liu et al.’s study, nurses considered physical demand to be the most important part of their mental workload, while mental demand was considered the least important [40]. Overall, we found that the scores for mental demand and frustration were the lowest, indicating that the HCWs responded in a positive manner to the pandemic.

A total of 89.14% of the participants in our study reported experiencing burnout, 31.46% of whom had moderate to high burnout. Previous studies have shown that the incidence of burnout among HCWs was up to 84.44% [55] and that the incidence of moderate to high burnout was 50.13% [56]. Burnout during the full relaxation of COVID-19 restrictions was much more prevalent but less severe among HCWs than before their relaxation. The incidence of burnout increased due to the increase in the number of infections caused by the full relaxation of the pandemic restrictions, but the reduction in severity was due to the improvement in the associated complementary supplies.

Burnout was associated with more adverse changes in physical and psychological health, quality of care and cost of care among HCWs. Therefore, early identification of the risk factors for burnout is essential. Increased fatigue can lead to burnout, as previous studies have confirmed [42]. Some studies have shown that an unmarried status is a protective factor against burnout [57]. However, married people had a lower risk of burnout in our study. Research from Çevik H and Ungan M suggested that this is due to improved social support [58]. In addition, Hu et al. noted that unmarried people had higher RPA scores [59], meaning that they may have higher expectations of their job and less experience, increasing their vulnerability to burnout.

The percentages of patients with EE, DP, and RPA scores above the cut-offs were 17.98%, 85.00%, and 20.22%, respectively. In the study by Galanis et al., the overall prevalence of EE was 34.1%, that of DP was 12.6%, and that of RPA was 15.2% [60]. Parola et al. reported that the prevalence of EE, DP, and RPA was 19.5%, 8.2%, and 9.3%, respectively [61]. In our research, the incidence of DP was much greater than that in previous studies. A high DP means an increased emotional gap between patients and HCWs, which is not conducive to establishing good professional relationships. However, the use of some isolation policies made high DP scores unavoidable. Studies have shown that some contact restrictions cause HCWs to become more isolated and emotionally distressed [62].

Previous studies have shown an association between fatigue and mental workload [39] and between burnout and mental workload [63]. In this study, we quantified the relationships among fatigue, mental workload, and burnout, which indicated that these factors could affect each other. This finding might alert managers to pay attention to all of these factors. That is, when assessing burnout among HCWs, fatigue and mental workload should also be accounted for.

This study has several limitations. First, this study investigated only the fatigue, mental workload and burnout of HCWs after the relaxation of strict zero-COVID-19 policies and failed to perform comparisons with the pre-epidemic and early epidemic phases to dynamically assess their changes. Second, although we explored the influences of fatigue, mental workload and burnout, we might have failed to identify all contributing factors. Third, we collected data mainly from HCWs in the surgery department. A broader sample may reveal differences among different departments and regions or beyond.

## Conclusion

Our study revealed a high prevalence of fatigue, mental workload, and burnout after the relaxation of the strict zero-COVID-19 policies. Fatigue, mental workload and burnout in HCWs were influenced by different factors, and these three factors were interrelated. This implies that when facing large-scale public emergencies, while the importance of comprehensive medical support cannot be overlooked, ultimately, attention needs to be given to the inner feelings of HCWs.

## Data Availability

The datasets generated and/or analysed during the current study are not publicly avaliable due the ethic of this study, but are available from the corresponding author upon reasonable request.
